# Effects of the Toxic Metals Arsenite and Cadmium on α-Synuclein Aggregation In Vitro and in Cells

**DOI:** 10.3390/ijms222111455

**Published:** 2021-10-24

**Authors:** Emma Lorentzon, Istvan Horvath, Ranjeet Kumar, Joana Isabel Rodrigues, Markus J. Tamás, Pernilla Wittung-Stafshede

**Affiliations:** 1 Department of Chemistry and Molecular Biology, University of Gothenburg, SE-405 30 Gothenburg, Sweden; emma.lorentzon@gu.se (E.L.); joana.rodrigues@gu.se (J.I.R.); 2 Department of Biology and Biological Engineering, Chalmers University of Technology, SE-412 96 Gothenburg, Sweden; istvanh@chalmers.se (I.H.); ranjeet@chalmers.se (R.K.)

**Keywords:** α-synuclein, amyloid formation, Parkinson’s disease, arsenic toxicity, cadmium toxicity

## Abstract

Exposure to heavy metals, including arsenic and cadmium, is associated with neurodegenerative disorders such as Parkinson’s disease. However, the mechanistic details of how these metals contribute to pathogenesis are not well understood. To search for underlying mechanisms involving α-synuclein, the protein that forms amyloids in Parkinson’s disease, we here assessed the effects of arsenic and cadmium on α-synuclein amyloid formation in vitro and in *Saccharomyces cerevisiae* (budding yeast) cells. Atomic force microscopy experiments with acetylated human α-synuclein demonstrated that amyloid fibers formed in the presence of the metals have a different fiber pitch compared to those formed without metals. Both metal ions become incorporated into the amyloid fibers, and cadmium also accelerated the nucleation step in the amyloid formation process, likely via binding to intermediate species. Fluorescence microscopy analyses of yeast cells expressing fluorescently tagged α-synuclein demonstrated that arsenic and cadmium affected the distribution of α-synuclein aggregates within the cells, reduced aggregate clearance, and aggravated α-synuclein toxicity. Taken together, our in vitro data demonstrate that interactions between these two metals and α-synuclein modulate the resulting amyloid fiber structures, which, in turn, might relate to the observed effects in the yeast cells. Whilst our study advances our understanding of how these metals affect α-synuclein biophysics, further in vitro characterization as well as human cell studies are desired to fully appreciate their role in the progression of Parkinson’s disease.

## 1. Introduction

Parkinson’s disease (PD) is the second most prevalent neurodegenerative disorder after Alzheimer’s disease (AD) and the most common movement disorder. PD is characterized by deterioration of motor neurons, predominantly dopaminergic neurons in *substantia nigra* [1], and the presence of neuronal inclusions, known as Lewy bodies (LBs). Amyloid fibers of the protein α-synuclein (αSyn) are the main component of LB pathology [2]. Other than PD, αSyn inclusions have been detected in a selection of neurodegenerative diseases, including LB variant of AD, multiple system atrophy, and dementia with Lewy bodies, among others [2,3]. Duplication and triplication of the αSyn gene *SNCA*, as well as several different missense mutations (A53T, A30P, A53E, H50Q, G51D, or E46K), have been identified in association with early onset of PD, suggesting that aberrant protein conformations and gene overexpression accelerate disease progression [4,5,6]. Clearly, αSyn amyloid formation is involved in PD development, but several modes of toxicity have been proposed, including the amyloids themselves, oligomeric intermediates, as well as αSyn interactions with for example lipid membranes [5,7]. A large part of the αSyn monomers present in a cell at normal conditions are either bound to lipid membranes or to chaperone proteins; thus, aberrant modulation of such interactions, and oligomer–lipid interactions, may play roles in the promotion of distinct features of PD, such as mitochondrial dysfunction and oxidative stress that are found along with amyloid formation [8,9,10]. 

**Figure 1:**
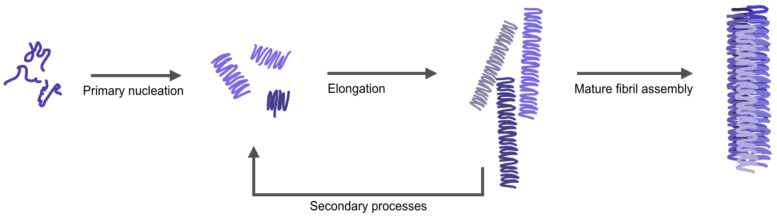
Schematic figure illustrating the different steps in αSyn aggregation; from monomers to oligomers via nucleation, elongating to single fibrils (protofibrils) that combine to larger amyloid structures of different morphologies. The single fibrils (and mature fibers) can also become the starting point for secondary processes, where both secondary nucleation and fiber fragmentation may occur.

Amyloid fibrils are often described as having a distinct morphology with different arrangements of twisted or parallel assemblies of thinner filaments [11]. Mature amyloid fibers form stepwise (Figure 1); starting with individual monomers of αSyn lumping together to form nuclei/oligomers (primary nucleation), these oligomers can bind additional monomers resulting in long individual fibers (elongation). Additionally, the process can be catalyzed by secondary processes; the surface of already formed fibers can work as a catalyst for the formation of new nuclei (secondary nucleation) and long fibers can break into shorter fragments creating new growing ends (fragmentation) [12,13]. Next, individual fibers can associate together to form thicker (mature) fibers. The assembly of thin fibers (often called protofilaments) into mature fibers gives rise to a periodicity in the fibrillar structure (denoted as pitch), which can be detected and measured by atomic force microscopy (AFM) or cryo-electron microscopy [14,15]. High resolution structures of αSyn fibrils, recently obtained by cryo-electron microscopy and solid state nuclear magnetic resonance (NMR) [16,17], have demonstrated that the observed pitch is created by intertwining of protofilaments (Table S1, for reported pitch values of αSyn amyloids). Variations in the arrangement of individual fibers relative each other, along with internal fiber differences, give rise to different fiber morphologies. External factors such as pH, ionic strength, and metals may affect the kinetics and pathways of αSyn amyloid formation, as well as the internal structure, pitch, and morphology of resulting fibers [18]. NMR data have suggested that differences in protein backbone conformation, salt bridges and electrostatic interactions between protofilaments relate with different polymorphs of αSyn amyloid fibers. This is important to study further as different morphologies of fibrils have been suggested to correlate with difference in cell toxicity [19].

Exposure to heavy metals has been linked to neurodegeneration [20,21,22]. Binding of metal ions to disease-associated proteins could not only affect their aggregation speed, but also change the resulting morphology of the fibrils. The interaction of metals with αSyn has been widely studied in recent years [23,24,25], and three main metal binding regions have been identified in the protein: the N-terminus, the histidine at position 50 (H50), and C-terminal residues. The N-terminus has been shown to be a high affinity Cu(I/II) site in WT αSyn [26,27]; however, it is important to note that the N-terminal acetylation of the protein blocks the Cu(II) binding capability from the N-terminal site [28]. H50 is the second binding site involved in Cu, and possibly Zn, coordination [26,29]. Lastly, within the acidic C-terminus, between residues 120 and 130, there are low affinity binding sites for several metal ions (Ca, Fe, Zn, Mn, Co, Ni). The effect of metals on the αSyn amyloid formation kinetics has also been investigated, and most metals promote its aggregation. However, most studies have focused on physiological metal ions and have been performed with mM concentrations of the metals in vitro. 

Humans are increasingly exposed to poisonous metals via intake of contaminated food and water. The heavy metal cadmium (Cd) and the metalloid arsenic (As) are clearly toxic and classified as human carcinogens [30,31]. Chronic heavy metal exposure, including As and Cd, has been implicated in promoting the progression of certain neurodegenerative and age-related disorders, including PD and AD [22,31,32,33,34]. However, the mechanisms by which As and Cd affect disease processes are still not fully understood. As and Cd each affect many processes in cells and can cause toxicity through several mechanisms, including interference with redox metabolism and induction of oxidative stress, impaired DNA repair, and inhibition of protein function and activity [35]. Importantly, it has been shown that As, in form of trivalent arsenite (As(III)) [36,37,38], and Cd(II) [39] induce widespread protein misfolding and aggregation in living yeast cells primarily by targeting nascent (i.e., not yet folded) proteins. As(III) and Cd(II) inhibit refolding of chemically denatured proteins in vitro [36,39,40,41], and both in vivo and in vitro data indicated that As(III)- and Cd(II)-triggered protein aggregates formed seeds that, in a gain-of-function mechanism, increased the misfolding and aggregation of other proteins [36,37,39]. Cd(II) has been shown to promote αSyn aggregation as well as the formation of αSyn oligomers in vitro [23,42], whilst As(III) caused a dose-dependent oligomerization of αSyn in a neuroblastoma cell line [43]. Given the profound effects of As(III) and Cd(II) on protein misfolding and aggregation as well as the suspected role of these metals in PD pathology, we here investigated their effects on αSyn amyloid formation in vitro and its aggregation in living yeast cells.

## 2. Results

The αSyn variant used in this study is the biologically relevant N-terminally acetylated αSyn (Ac αSyn), as acetylation has been reported to be a general posttranslational modification to the protein [44], which also occurs naturally when expressed in yeast [45]. Moreover, the N-terminus of αSyn without acetylation may engage in additional metal binding *in vitro*, which is not biologically relevant and is not investigated in this study. 

### 2.1. Effects of As(III) and Cd(II) on Ac αSyn Amyloid Fibril Morphology 

**Figure 2:**
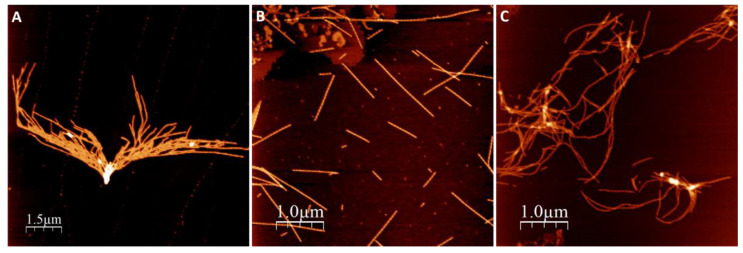
AFM images of amyloid fibrils formed after 90 h aggregation in pH 7.4. (**A**) Ac αSyn, (**B**) Ac αSyn + NaAsO_2_ 1:10, (**C**) Ac αSyn + CdCl_2_ 1:10.

We first investigated amyloid fibril morphology of αSyn amyloids after the protein had been incubated in the presence of NaAsO_2_ and CdCl_2_. In these experiments, 50 µM Ac αSyn was mixed with 500 µM metal ions (the concentration giving the largest change to Ac αSyn aggregation kinetics, see below), and then incubated at 37 °C with agitation for 3 days at pH 7.4. The resulting fibril morphology was analyzed by atomic force microscopy (AFM) (Figure 2).

**Figure 3:**
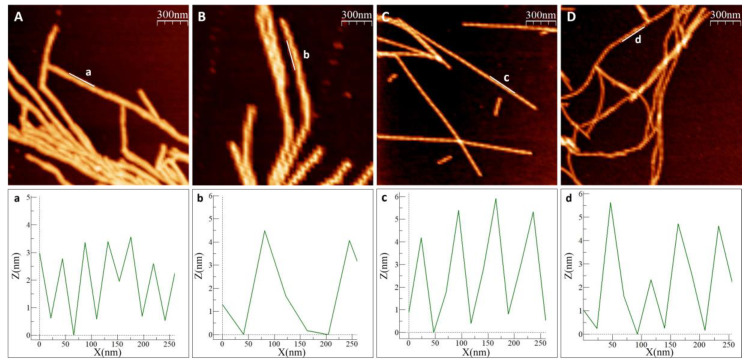
Measurement of fiber pitch in AFM images. (**A**,**B**) Ac αSyn with two distinct pitch types, (**a**) is showing the shorter pitch, (**b**) the longer pitch, (**C**) Ac αSyn + NaAsO_2_ 1:10, (**D**) Ac αSyn + CdCl_2_ 1:10. The pitch was measured as the distance between two maxima in the height curves. Heights are tracked along the indicated lines (**a**–**d**).

In all experiments, with and without metals, we observed amyloid fibrils with heights of 6–8 nm. Visual inspection of the AFM images revealed different amyloid appearances: straight fibrils with As(III), and curved fibrils with Cd(II), while for αSyn alone (no metal added) we detected mostly short fibrils lumped together. Higher magnification images of individual fibers (Figure 3) show a twisting pattern along the fibers, which corresponds to the fiber pitch. When Ac αSyn was aggregated without metals, we identified two sizes of fibril pitch (found in different fibers, but both kinds present in the same sample): one with pitch of 49 nm and the other one with 104 nm. Interestingly, for Ac αSyn amyloids formed in the presence of As(III) or Cd(II), samples were homogeneous with only one type of fibril morphology identified—with 71 nm pitch for As(III) and 80 nm pitch for Cd(II) (Table 1). There are various values reported for the pitch of αSyn or Ac αSyn fibrils (without metals) that cover the range of 40–120 nm (summarized in Table S1), and thus it appears that these values are highly sensitive to the experimental aggregation conditions. Nevertheless, the observed differences in αSyn amyloid pitch with and without As(III) and Cd(II) at our conditions suggest that the metals modulate protofilament interactions within the mature fiber.

To assess if metals are incorporated in the resulting αSyn amyloid fibers, we determined the metal concentration in the amyloids after 5 days of aggregation with 1:1 protein to metal samples. The metal content in the fibers were indirectly determined from analysis by inductively coupled plasma mass spectrometry (ICP-MS) of metal content in the solution after αSyn amyloids had been removed by centrifugation. In these experiments, a reduction of more than 50% of added metal ion concentration was detected in the remaining solution after the fibers had been spun down (Table S2). Thus, As(III) and Cd(II) ions are being incorporated into the αSyn amyloid fiber structures with around 1-to-2 metal-to-monomer stoichiometry. 

### 2.2. In Vitro Ac αSyn Amyloid Formation Kinetics in the Presence of Cd(II) and As(III)

**Figure 4:**
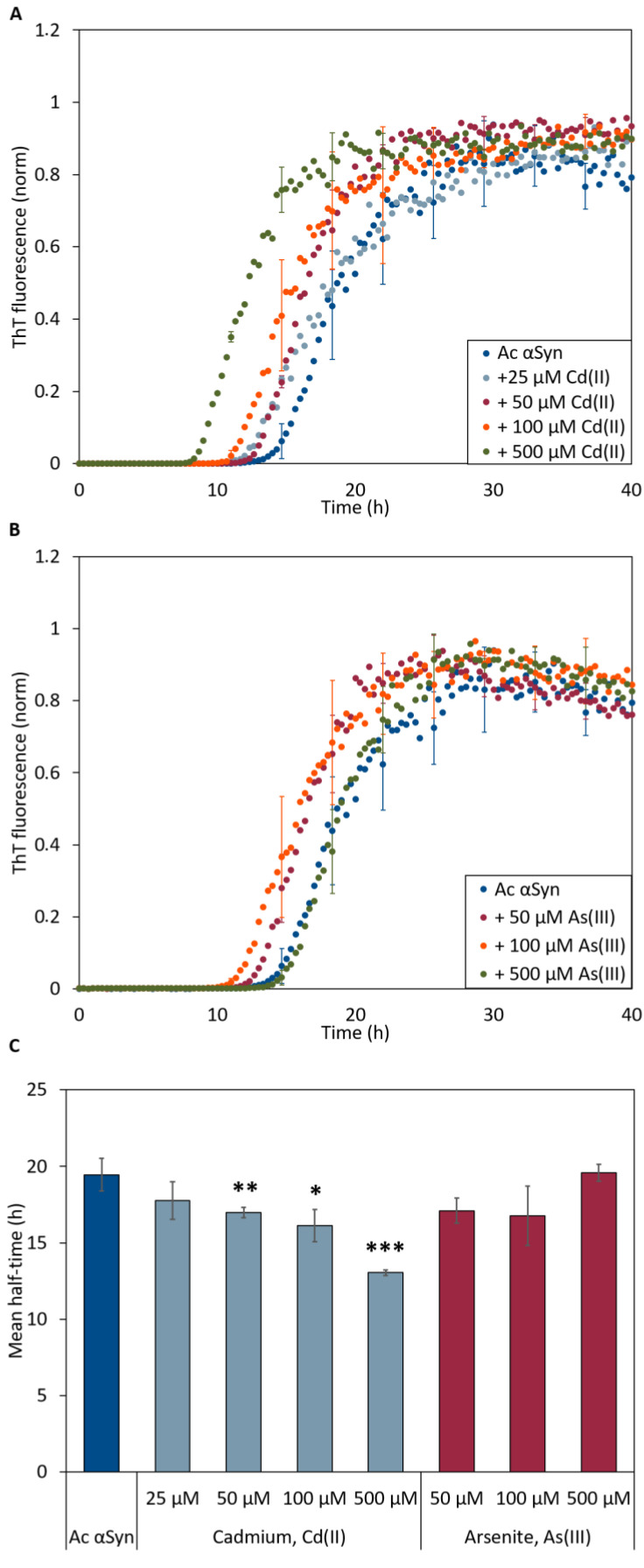
ThT fluorescence curves for aggregation of 50 μM Ac αSyn in the presence of (**A**) Cd(II) and (**B**) As(III)-ions at increasing concentrations at pH 7.4. The curves in (**A**,**B**) are representative kinetic traces based on the averages of three technical replicates. (**C**) Aggregation curve midpoints (i.e. ThT fluorescence at 0.5) derived from ThT-detected kinetic data in (**A**,**B**), for various additions of Cd(II) (light blue) and As(III) (red) to Ac αSyn. Error bars represent SD from three independent replicates with three to four mechanical replicates each. Significance scale (different relative to the value for protein without metals) according to: * *p* < 0.05, ** *p* < 0.01, and *** *p* > 0.005.

To address whether the metal ions also affect amyloid formation kinetics, we turned to time-resolved in vitro aggregation assays. We monitored the kinetics of amyloid formation using the Thioflavin T fluorescence (ThT) assay with 50 µM Ac αSyn as a function of NaAsO_2_ and CdCl_2_ (1:1 to 1:10 protein-to-metal ratio) concentrations at 37 °C under agitation with a glass bead at pH 7.4. The aggregation of Ac αSyn without added metals followed a sigmoidal trace similar to that for wild type αSyn [14,46] with a lag time of ~12 h and aggregation was completed by 30 h. In the presence of Cd(II), we observed metal concentration-dependent decrease of the lag time for Ac αSyn amyloid formation (down to ~7 h at the highest metal concentration) (Figure 4A,C). In the case of As(III), we detected apparent shorter lag times for 50 and 100 µM metal ions; however, the effect was not significantly different as compared to the aggregation kinetics of Ac αSyn alone (Figure 4B,C).

Because these experiments may include all processes in Figure 1, we next investigated the fibril elongation step specifically using fiber-seeded experiments to bypass primary nucleation. Here, we followed the aggregation of Ac αSyn when fiber seeds (10 molar %) were added to the monomeric protein (Figure S1). Monomers in the presence of seeds can directly bind to the ends of the pre-made fibers, and indeed, what we observe from seeded aggregation is an immediate increase in ThT intensity at time zero for all samples and the presence or absence of metals had no effect on the kinetics. We thus conclude that neither As(III) nor Cd(II) has any significant impact on the elongation step in the Ac αSyn amyloid formation reaction. The overall effect on the process observed for Cd(II) in Figure 4 thus implies that this metal mainly affects primary nucleation. 

Since Cd(II) appears to affect the primary nucleation step, the metal may bind to either αSyn monomers or early assembled intermediates. Therefore, we searched for potential interactions between the metals and monomeric Ac αSyn using isothermal titration calorimetry. Monomeric Ac αSyn was added to the reaction cell and the metal ions were injected stepwise to the cell in up to six times molar excess. Figure S2 demonstrates a lack of heat changes during the titration, implying no direct binding between αSyn Ac monomers and As(III). In the case of Cd(II), heat change was observed for the first few titrations, suggesting a possible interaction between the metal and a small fraction of contaminating αSyn oligomers. Even if we always purify αSyn to start with pure monomer samples in each experiment, a small fraction of protein will with time start to assemble. If Cd(II) would bind to monomeric αSyn, we would have detected heat changes that saturated at one to one or higher metal to protein ratios. Unfortunately, the acquired dataset was not adequate for quantifying the ΔH and Ka values of the interaction.

### 2.3. As(III) and Cd(II) Affect αSyn Aggregate Distribution within Yeast Cells

To bring the acquired in vitro results to an in vivo setting, we used *Saccharomyces cerevisiae* (budding yeast) as an established cellular model for αSyn biology [47]. It has previously been shown that αSyn fused to green fluorescent protein (αSyn-GFP) expressed in yeast first localizes to the plasma membrane (1–2 h after induction of expression, one can see a characteristic “halo” on the cell surface), then small aggregates appear at the plasma membrane and, upon an extended expression, large aggregates are visible in the interior of the cell [48]. We expressed αSyn-GFP from the inducible *GAL1*-promotor in living yeast cells and quantified aggregates of αSyn as fluorescent foci associated with the cell surface/plasma membrane and in the interior of the cell. We counted cells that had 1–2 aggregates/cell and those with ≥3 aggregates/cell in the absence and presence of As(III) and Cd(II). The metal concentrations used in the yeast assays (500 µM NaAsO_2_ and 50 μM CdCl_2_) do not affect cell viability, aggregate dissolution, or clearance mechanism during the experimental time course [36,39]. 

**Figure 5:**
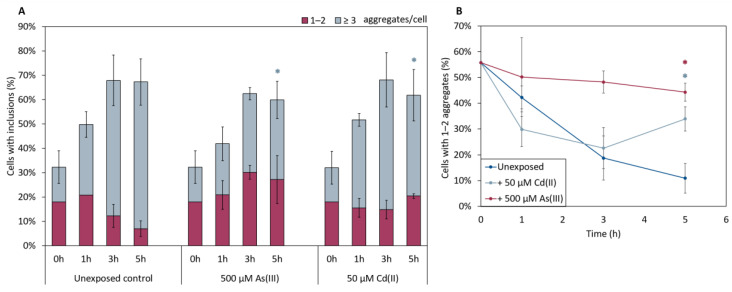
(**A**) In vivo aggregation of αSyn in metal exposed yeast cells. The αSyn-GFP localization was monitored in wild-type cells before and after 1, 3, and 5 h of exposure to either 500 µM NaAsO_2_ or 50 µM CdCl_2_. The fractions of cells containing aggregates (either 1–2 or ≥3 aggregates/cell) were determined by visual inspection of approx. 300 cells per condition. The error bars represent SD from three independent biological replicates. Significance is calculated with the unexposed cells as reference, (un-paired two-tailed student’s *t*-test, blue: ≥3 aggregates/cell; * *p* < 0.05). (**B**) Percentage of cells with 1–2 aggregates/cell out of the total percentage of cells with αSyn-GFP inclusions. Significance here refers to 1–2 aggregates/cell, red: 500 µM NaAsO_2_; light blue: 50 µM CdCl_2_, with * *p* < 0.05.

**Figure 6:**
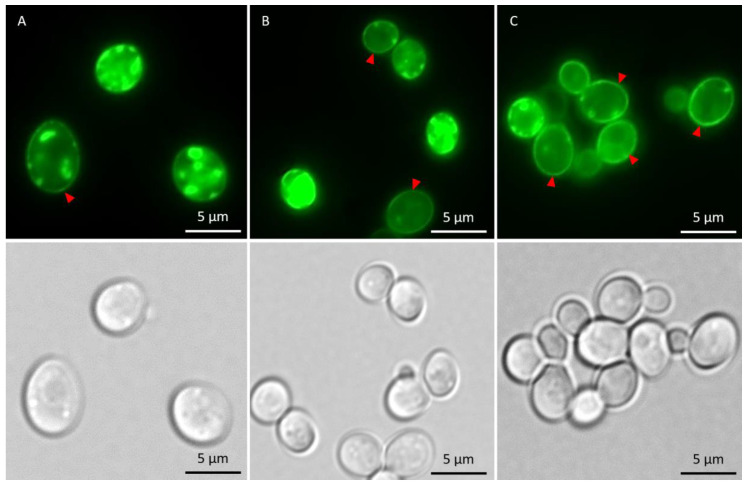
Fluorescent microscopy images showing αSyn-GFP in the cells in the absence of metal and after 5 h of metal stress. Cells with membrane-associated αSyn-GFP are indicated with red arrows. (**A**) αSyn-GFP cells without metal exposure. (**B**) αSyn-GFP cells + 50 µM CdCl_2_. (**C**) αSyn-GFP + 500 µM NaAsO_2_. The GFP-foci/aggregates were determined by visual inspection of 200 to 300 cells per condition and time point. Representative images are shown.

In the absence of metals, the total fraction of cells having αSyn aggregates increased after induction of αSyn-GFP expression, and the proportion of cells with ≥3 αSyn-GFP aggregates/cell increased during the course of the experiment (Figure 5). The kinetics of aggregate formation and the total αSyn aggregation level was not significantly affected in cells exposed to As(III) or Cd(II); however, the distribution of cells with 1–2 aggregates/cell and ≥3 aggregates/cell was affected. In the absence of metals, most cells (~90%) had ≥3 αSyn-GFP aggregates/cell at the 5 h time point. In contrast, exposure to As(III) and Cd(II) resulted in a larger proportion of cells with 1–2 aggregates/cell; the proportion of cells with 1–2 aggregates/cell was ~10% in unexposed cells whilst it was ~45% and ~35% in As(III) and Cd(II) exposed cells, respectively (Figure 5A,B). We also noted that As(III) and Cd(II) exposed cells retained substantially more αSyn-GFP in or near the plasma membrane than unexposed cells (Figure 6; red arrows). Approximately 37% (±6%) of un-stressed cells containing aggregates had a visible “halo” of αSyn-GFP in the plasma membrane, cells exposed to Cd(II) had 44% (±4%), compared to the cells exposed to As(III) that had 73% (±2.3%) of aggregate-containing cells having plasma membrane-associated αSyn-GFP. Hence, As(III) and Cd(II) affect αSyn-GFP localization in vivo.

### 2.4. Impact of As(III) and Cd(II) on Newly Synthesized αSyn, αSyn Clearance, and αSyn Toxicity in Yeast Cells

**Figure 7:**
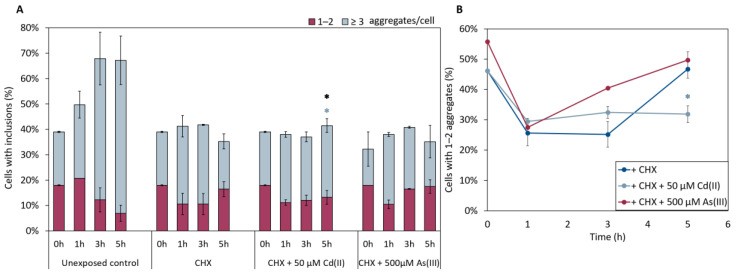
(**A**) In vivo aggregation of αSyn-GFP in the presence of 0.1 mg/mL of the protein synthesis inhibitor cycloheximide (CHX). αSyn-GFP foci localization was monitored after 0, 1, 3, and 5 h of exposure to either NaAsO_2_ or CdCl_2_. The fractions of cells containing aggregates (either 1–2 or ≥3 aggregates/cell) were determined by visual inspection of approx. 300 cells per condition. The error bars represent SD from three independent biological replicates. Significance is calculated with the untreated control as reference (un-paired two-tailed student’s *t*-test against CHX-treated cells, black: total fraction of cells with aggregates; blue: ≥3 aggregates/cell; * *p* < 0.05). (**B**) Percentage of 1–2 aggregates out of the total percentage of cells with αSyn-GFP inclusions when CHX is present in the cells. Significance here refers to 1–2 aggregates/cell, light blue: 50 µM CdCl_2_, with * *p* < 0.05.

We next examined whether As(III) and/or Cd(II) targets nascent (polypeptides made by ribosomes after metal additions) or mature (polypeptides synthesized prior to metal additions) αSyn. For this, we treated cells expressing αSyn-GFP with As(III) or Cd(II) and simultaneously added the protein synthesis inhibitor cycloheximide (CHX), and monitored the number of αSyn-GFP aggregates over time. CHX addition inhibited αSyn aggregation in equal measure for untreated and metal exposed cells (Figure 7), suggesting that the metals primarily affect nascent αSyn and not protein already present in the cells.

**Figure 8:**
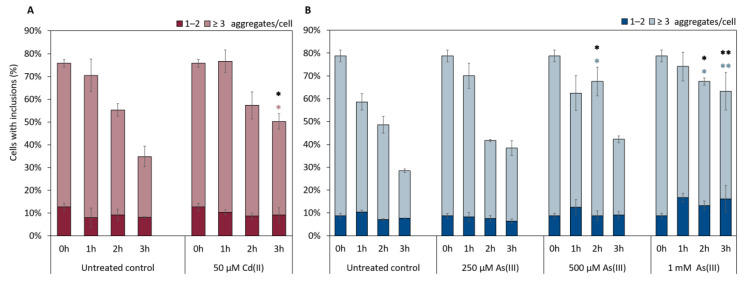
Clearance of αSyn-GFP aggregates (1–2 aggregates, or ≥3) in vivo over 3 h in the presence or absence of NaAsO_2_ or CdCl_2_. αSyn-GFP expression was induced for 5 h (Gal-induction), then induction was stopped (addition of glucose) and aggregate clearance monitored in the absence and presence of (**A**) 50 µM CdCl_2_ and (**B**) 250 µM, 500 µM, and 1 mM NaAsO_2_. The percentage of cells containing aggregates was determined by visual inspection of approx. 300 cells per condition and time point. The error bars represent SD from three independent biological replicates. * *p* < 0.05 and ** *p* < 0.01 compared with the untreated control (un-paired two-tailed student’s *t*-test, black: total fraction of cells with aggregates; (**A**) pink or (**B**) light blue: ≥ 3 aggregates/cell).

Clearance of αSyn-GFP aggregates involves internalization through endocytosis and subsequent degradation through autophagy and vacuolar degradation [49,50]. Given the observed effects of As(III) and Cd(II) on αSyn-GFP aggregate localization/distribution (Figure 6 and Figure 7), we next asked if these metals affect αSyn clearance. For this, we first induced the expression of αSyn-GFP from the *GAL1* promoter during 5 h, then turned off αSyn-GFP expression and quantified aggregate levels as above. As shown in Figure 8, As(III) appears to inhibit αSyn aggregate clearance in a concentration-dependent manner. Likewise, Cd(II) at the concentration used (higher concentrations are too toxic to cells and could not be tested here) affected αSyn clearance. 

**Figure 9:**
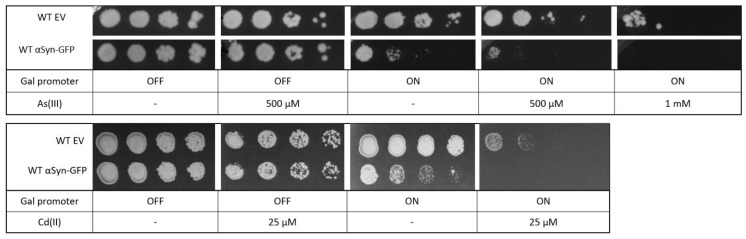
Yeast growth assays. BY4741 cells were transformed with an empty vector or a plasmid expressing αSyn-GFP. Cells were grown overnight in YNB-URA with 2% glucose as carbon source. Cell cultures were adjusted to the same OD, serially diluted, and spotted into YNB-URA with 2% glucose or 2% galactose as carbon source and the indicated As(III) and Cd(II) concentrations. Plates were incubated at 30 °C for 3 days. Images shown are representative of three biological replicates.

αSyn has been shown to be toxic to yeast cells [48], and we next addressed whether yeast expressing αSyn-GFP are more susceptible to metal stress as well. Yeast cells containing αSyn-GFP were grown overnight in a glucose medium (αSyn-GFP expression off) and then spotted onto agar plates with galactose (αSyn-GFP expression on) or without galactose (αSyn-GFP expression off) as well as different concentrations of As(III) and Cd(II). Cells expressing αSyn were somewhat more sensitive to both As(III) and Cd(II) than cells with the corresponding empty plasmid or cells in which αSyn-GFP expression is off (Figure 9). Thus, the presence of metals aggravates αSyn toxicity in yeast. 

## 3. Discussion

The in vitro data suggested that As(III) and Cd(II) interaction with αSyn changes the resulting structures of the amyloid fibers, at least with respect to the fiber pitch. Our AFM clearly showed that both As(III) and Cd(II) change the fiber structural characteristics of Ac αSyn when the metals were added in a 10-fold molar excess. The change in pitch values and the fact that the samples are more homogenous (containing one type of fiber) suggest that amyloid formation in the presence of these metals affect fiber morphology (at least with respect to pitch and heterogeneity). Our ICP-MS results further indicate that both As(III) and Cd(II) are present in the fibers; this can occur either by the metals becoming bound to the surface of protofibrils or by being incorporated into the protofibrils at earlier stages of the aggregation process, giving rise to the altered mature fibril arrangement. Binding of metals to mature fibers has been reported earlier in a study by Dearborn et al. [51] where dense threads of metal ions on the surface of αSyn fibers were identified using electron microscopy. Additionally, the pitch for the αSyn fibers in that study (77 nm) is similar to what we have found for the As(III) and Cd(II) containing conditions here. Interestingly, a decrease in the fiber pitch values has been reported for various pathological αSyn core mutants [15,52,53] and also upon truncation of the αSyn C-terminal domain [54] (see Table S1). 

Based on the similarity with these studies, As(III) and Cd(II) might interact with either the core or the C-terminus of αSyn. For Cd(II), a possible candidate site for interaction is histidine 50 located in the amyloid core region. H50 is linked to the pathological mutation H50Q and coordination of Cd(II) to this site might affect the protofilament-surface thereby affecting the assembly and thus periodicity of mature fibers. As(III) preferentially binds to cysteine residues but non-thiol binding (e.g., lysine) might also occur [55]. The latter likely occurs in the case of αSyn, which lacks cysteine. Both As(III) and Cd(II) might also bind in the C-terminal domain of αSyn where weak binding sites (negatively charged residues) for various cationic metals have been identified [25]. It is unclear how the conformation of the C-terminal domain is affecting the overall fiber morphology, but as mentioned above, the removal of the C-terminal domain results in fibers with shorter pitch. Taken together, although the binding sites are not identified, binding of these metals to αSyn affect the periodicity of mature fibers. 

The aggregation assays further support the notion that Cd(II) interacts with Ac αSyn: the decrease in lag time and midpoint would imply that the primary nucleation step is promoted by the metal since the critical concentration of fibril forming nuclei is reached at an earlier time point. Notably, we observed a statistically significant decrease in lag time and midpoint upon addition of 50 M CdCl_2_, which is the same concentration as used in the in vivo experiments. This effect implies that the metal ion binds during early stages of amyloid formation, which can be achieved either by the metal binding to protein monomers and promoting oligomer formation, or by binding to oligomers and stabilizing them in their process towards amyloid fibers. The results from the ITC does not support monomer interactions but instead imply that Cd(II) interacts with oligomeric Ac αSyn species. Since the ITC is performed under constant stirring, a small amount of oligomers may form in the sample and they may bind the titrated Cd(II) ions and give rise to the initial heat changes. In terms of As(III), it did not affect have a significant effect on amyloid formation kinetics, but it may still bind to αSyn at any stage of the aggregation process or to the fibers after their formation (but before maturation to affect fiber pitch). 

Our in vivo assays show that As(III) and Cd(II) affect αSyn-GFP localization, clearance, and toxicity in yeast cells. However, the mechanism(s) by which metals impact αSyn biology remain to be understood. It is possible that metal interactions with αSyn and the resulting effects on the amyloid fiber morphology seen in vitro, underlie the observed effects (such as the number of aggregates, localization, clearance, and toxicity) when αSyn was expressed in yeast cells. Metals may interfere with protein function by direct binding to functional groups, by displacing essential metal ions in metalloproteins, or by catalyzing oxidation of amino acid side chains [33,56]. Alternatively, As(III) and Cd(II) could disturb other processes in vivo that in turn indirectly affect αSyn biology and thereby cell toxicity. For example, metal inhibition of endocytosis, autophagy, and vacuolar degradation processes might impact αSyn clearance and toxicity. Similarly, both As(III) and Cd(II) induce widespread protein aggregation in cells [36,39], which might shift the available pool of molecular chaperones away from αSyn [57] to other misfolded and aggregated proteins. This, in turn, might result in a decreased capacity to protect the cell against αSyn aggregation and toxicity. Finally, As(III) and Cd(II) exposure can induce oxidative stress and disrupt ATP generation [22], which in turn might also impact αSyn biology.

Different forms of αSyn aggregate species exhibit different toxicities in vivo, with more and more evidence suggesting that oligomers are more toxic than fully formed amyloid fibers [19]. We observed that more αSyn is retained in the plasma membrane during As(III) and Cd(II) exposure, and hypothesize that metal binding to αSyn might cause the protein to stay longer in the membrane as monomers and oligomers, thereby contributing to toxicity during long-term metal exposure. Future in vitro studies should investigate the effects of As(III) and Cd(II) on αSyn aggregation in the presence of lipid vesicles to mimic membrane interactions. Metals may also affect the size and properties of aggregates. Indeed, we recently showed that Cd(II)-induced luciferase aggregates are smaller than heat-induced luciferase aggregates and that Cd(II)-aggregated proteins form seeds that increase the misfolding of other proteins [39]. It will be important to determine whether metals directly interact with αSyn in vivo, and whether such interactions contribute to the here observed alterations in toxicity. In any case, metal interaction is likely to occur primarily with nascent αSyn since inhibition of protein synthesis by CHX prevented αSyn aggregation in vivo. 

**Figure 10:**
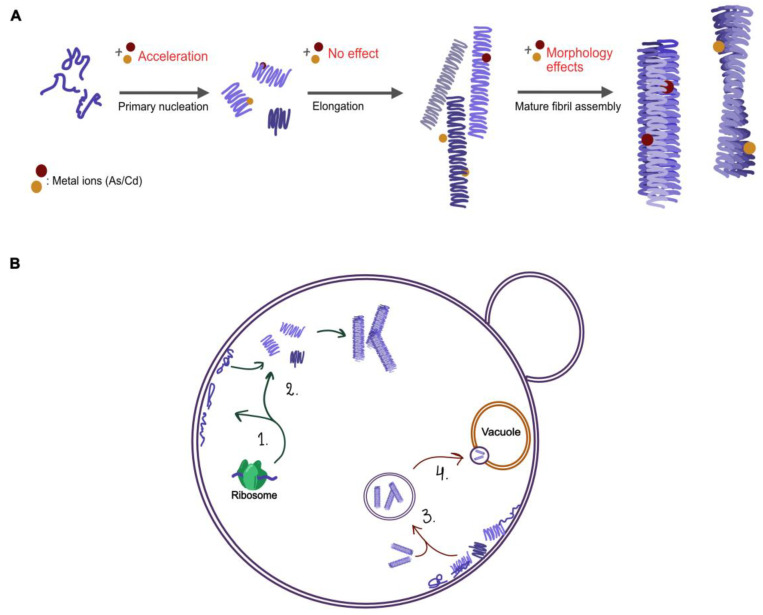
Illustration of the possible pathways in αSyn aggregation (**A**) and processes of αSyn in yeast (**B**) that might be affected by As(III) and Cd(II). (**A**) In vitro addition of Cd(II) accelerates the primary nucleation step; possible changes to the internal structure of single fibrils result in morphological changes to the mature amyloid fibers. (**B**) (1–2) the synthesis of αSyn and its interaction with plasma membranes, (3) the localization and relocation of aggregates, and (4) clearance of αSyn aggregates.

To sum up, chronic arsenic and cadmium exposure is associated with a variety of neurodegenerative disorders caused by aberrant protein folding including PD and AD [31,32,33,58,59]. Our data presented here indicates that As(II) and Cd(II) interactions with αSyn intermediate assemblies change the resulting conformation of the amyloid fibers in vitro and that As(III) and Cd(II) affect αSyn biology and toxicity in vivo (see summary Figure 10). The effects of As(III) and Cd(II) on protein misfolding in general and on αSyn conformation in particular, might explain how these metals contribute to disease. Nevertheless, further in vitro characterization as well as human cell studies are needed to fully understand the effects of these metals on αSyn aggregation and on their role in PD pathology. 

## 4. Methods

### 4.1. Expression and Purification of αSyn

The protein Ac αSyn was expressed and purified as previously described [46].

### 4.2. ThT Aggregation Assay

The amyloid formation assays were conducted using 96-well half-area transparent bottom plates (CLS3881; Corning, Corning, NY, USA), with 2 mm glass bead in each well in a plate reader-incubator instrument (Fluostar Optima; BMG Labtech, Ortenberg, Germany). To prevent contamination and evaporation, the plates were sealed with transparent tape. Measurements were performed in TBS (0.05 M Tris-HCl buffer, pH 7.4 with 0.15 M NaCl, Sigma-Aldrich, St. Louis, MO, USA) in the presence of 20 µM Thioflavin T (ThT; T3516; Sigma-Aldrich, St. Louis, MO, USA) at 37 °C using 5 min 200 rpm agitation in the beginning of each 20 min measurement cycle. Fluorescence was measured from the bottom of the plate. The metal salts sodium arsenite (NaAsO_2_) and cadmium chloride (CdCl_2_) was added to the samples at the indicated concentrations. The protein was gel filtered before each experiment to ensure that the starting product was monomers at a concentration of 50 µM. The samples were incubated for 70–90 h with measurement every 20 min; ThT was exited at 440 nm and emission recorded at 480 nm. For the seeded aggregation, seeds were obtained by incubating Ac αSyn under the conditions described above. After the aggregation was complete, the samples were sonicated for 10 s using a probe sonicator (stepped microtip and Ultrasonic Processor Sonics Vibra-Cell; Sonics & Materials, Newtown, CT, USA) at an amplitude of 20%, and an alternating cycle of 5 s on and 5 s off. The resulting short fibrils were added to monomeric protein in the seeding experiments where the measurement procedure did not include addition of glass beads and agitation of the samples in order to avoid breaking of the growing fibers. All experiments were performed with 4–5 replicates and repeated three independent times. 

### 4.3. AFM

Samples from the ThT aggregation assays at 90 h were diluted 10 times in MilliQ water and deposited onto freshly cleaved mica. After 15 min, the mica samples were rinsed twice with MilliQ water then dried completely under a gentle stream of nitrogen. The images were captured using an NTEGRA Prima setup (NT-MDT, Moscow, Russia) at a resonance frequency around 180 kHz, using a gold-coated single crystal silicon cantilever (NSG01, spring constant of ~5.1 N/m; NT-MDT, Moscow, Russia). Images of 512 pixels were captured at a scan rate ranging from 0.3–0.5 Hz, scanning a minimum of four 50 µm × 50 µm areas in each sample. Images were analyzed using WSxM 5.0 software [60], with around 60 fibers measured per condition. The images shown in this article are representative for each condition.

### 4.4. ITC

Measurements were performed using a Microcal iTC200 calorimeter (Malvern Instruments, Malvern, UK), with a sample cell volume of 206 μL and titration syringe of 40 μL. Prior to the experiments Ac αSyn was gel filtered on BioRad Enrich 70 column using TBS as running buffer and the metal salts NaAsO_2_ and CdCl_2_ were dissolved in the same buffer. The temperature was set to 25 °C, initial delay 120 s, reference power 6 μCal/s, with 750 rpm stirring, and high feedback/gain was used. Titrations of 2 μL of As(III) or Cd(II) salts (up to 6 times molar excess) to Ac αSyn (50 µM) were performed with 4 s duration, spacing of 180 s, and filtering time of 2 s. For all titration series, a first aliquot of 0.4 μL was injected but not included in the analysis. The heat change during the titration was registered in real time, and peaks were integrated to obtain the heat change per mole of injectant, using the Origin software (OriginLab, Northampton, MA, USA) supplied with the instrument. 

### 4.5. Metal Concentration in Fibers

The concentration of metal bound in fibers was measured by aggregating the protein with the metal salts NaAsO_2_ and CdCl_2_, at a protein-to-metal ratio of 1:1, over three days at 37 °C and 200 rpm, with a 2 mm glass bead in the sample tubes. The samples were spun down at 20 k rpm for 25 min and the supernatant collected for analysis. The content of As(III) and Cd(II) in the samples was measured using an inductively coupled plasma-mass spectrometry (ICP-MS) as previously described [38]. 

### 4.6. Yeast Strains and Growth Conditions

The *S. cerevisiae* strain used in this study was BY4741 WT (*MATa his3Δ1 leu2Δ0 met15Δ0 ura3Δ0*). Plasmids used were the following: pRS426-GAL [61] and pRS426-GAL-αSynWT-GFP [47]. Cells were cultivated in minimal YNB (yeast nitrogen base) medium lacking appropriate amino acid for selection, and 2% glucose, raffinose or galactose as carbon source. Stock solutions of sodium arsenite (NaAsO_2_), cadmium chloride (CdCl_2_), and cycloheximide (CHX) (all from Sigma-Aldrich, St. Louis, MO, USA) were added directly to the growth medium for the florescence microscopy and growth assays at the indicated concentrations.

### 4.7. Fluorescence Microscopy

To follow the aggregation of αSyn in the cells, transformants containing αSyn-GFP (GAL-promotor) were grown in YNB medium lacking uracil (YNB-Ura; Formedium, Hunstanton, UK) and 2% glucose overnight. The culture was then diluted to an optical density (OD_600nm_) of 0.2 and the medium changed to YNB-Ura with 2% raffinose, and grown to an OD of 0.5–0.6 overnight. To promote expression of αSyn-GFP, the media was changed to contain 2% galactose, and the culture induced for 2 h to ensure that the cells had started to express measurable levels of αSyn-GFP before sample collection. 

### 4.8. Growth Assays on Plate

Cells were grown in YNB lacking uracil (YNB-Ura; Formedium, Hunstanton, UK) overnight. The OD of the cultures was adjusted to the same value (0.8–1), serially diluted, and spotted onto YNB-Ura agar plates with 2% glucose or 2% galactose as carbon source, containing the indicated As(III) and Cd(II) concentrations. Plates were incubated at 30 °C and monitored for 2–3 days. Representative pictures are shown from at least 3 biological repetitions.

## Figures and Tables

**Table 1:** Mean pitch of Ac αSyn amyloid fibrils at pH 7.4 from AFM data. Ac αSyn aggregating alone gives rise to two types of fibers: fibrils with short pitch and fibrils with a longer pitch. The values are a result of 50–60 measured fibers and significance calculated using Welch’s two-sided *t*-test (with respect difference to the 49 nm value). Significance according to *** *p* < 0.001.

Fiber Type	Height	Mean Pitch (nm)	SD
**Ac αSyn**	6–8 nm	49	8.8
	6–8 nm	104 ***	24.9
**Ac αSyn + 500 µM NaAsO_2_**	6–8 nm	71 ***	18.9
**Ac αSyn + 500 µM CdCl_2_**	6–8 nm	80 ***	11.5

## Data Availability

All data generated and analyzed during this study are included in this article and its Supplementary Information.

## Supplementary Materials

The following are available online at https://www.mdpi.com/article/10.3390/ijms222111455/s1.
