# Supplementary material for: Effects of the Toxic Metals Arsenite and Cadmium on α-Synuclein Aggregation In Vitro and in Cells
Source: Int J Mol Sci. 2021 Oct 24;22(21):11455. doi: 10.3390/ijms222111455 (PMC8584132; doi:10.3390/ijms222111455)
Supplement: Supplementary file 1 [file ijms-22-11455-s001.zip › ijms-1387038-supplementary.pdf]

Supplementary Information

Effects of the toxic metals arsenite and cadmium on α-synuclein aggregation *in vitro* and in cells

Emma Lorentzon[1], Istvan Horvath[2], Ranjeet Kumar[2], Joana Isabel Rodrigues[1], Markus J Tamás[1], Pernilla Wittung-Stafshede[2]

[1]*Department of Chemistry and Molecular Biology, University of Gothenburg, SE-405 30 Gothenburg, Sweden*

[2]*Department of Biology and Biological Engineering, Chalmers University of Technology, SE-412 96 Gothenburg, Sweden*

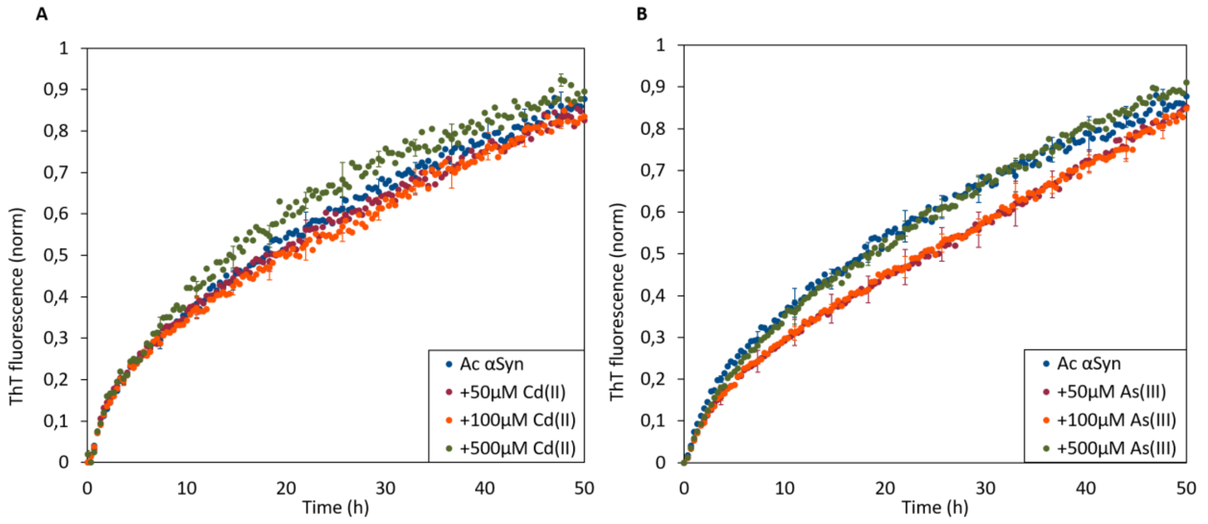

Figure S1 - ThT fluorescence kinetic traces for 50µM Ac αSyn aggregation (pH 7.4) in the presence of 10% amyloid fibre seeds. The effect of the metals is no longer visible.

Table S1 – Reported values for αSyn amyloid fibril pitch based on AFM and cryo-EM measurements.

Reference	Protein	Method	Pitch
Li Y. et al., 2018 [1]	WT αSyn	CryoEM	119 nm
R. Guerrero-Ferreira et al., 2019 [2]	WT αSyn	CryoEM	108 nm
Li B. et al., 2018 [3]	WT αSyn	CryoEM	rod: 92 nm<br>twister: 46 nm
Ni X. et al., 2019 [4]	Ac αSyn	CryoEM	121 nm
Ni X. et al., 2019 [4]	Ac αSyn1-122	CryoEM	85 nm
Ni X. et al., 2019 [4]	Ac αSyn1-103	CryoEM	65 nm
Zhao K. et al., 2020 [5]	Ac αSyn	AFM	120 nm
Zhao K. et al., 2020 [5]	Ac αSyn E46K	AFM/CryoEM	64 nm
Iyer A. et al., 2016 [6]	Ac αSyn	AFM	115 nm
Boyer D. et al., 2019 [7]	α Syn H50Q	CryoEM	90 nm
Sun Y. et al., 2020 [8]	Ac αSyn A53T	CryoEM	97 nm

Table S2 - Measured values of As(III) and Cd(II) concentrations in ppb, measured using ICP-MS. Samples (1 to 1 protein to metal) were spun down (to get amyloids at the bottom) after no incubation and after 5 days of aggregation, followed by metal analysis of the supernatant. The 'missing' metal from the solution (comparing 0 to 5 days) must have been incorporated into the amyloids.

**As^{3+}**	**Conc. (µM)**	**Cd^{2+}**	**Conc. (µM)**
AVG As(III)0 h	50	AVG Cd(II) 0 h	50
AVG As(III) 5 days	20	AVG Cd(II) 5 days	24
Left in solution (%):	40%		49%

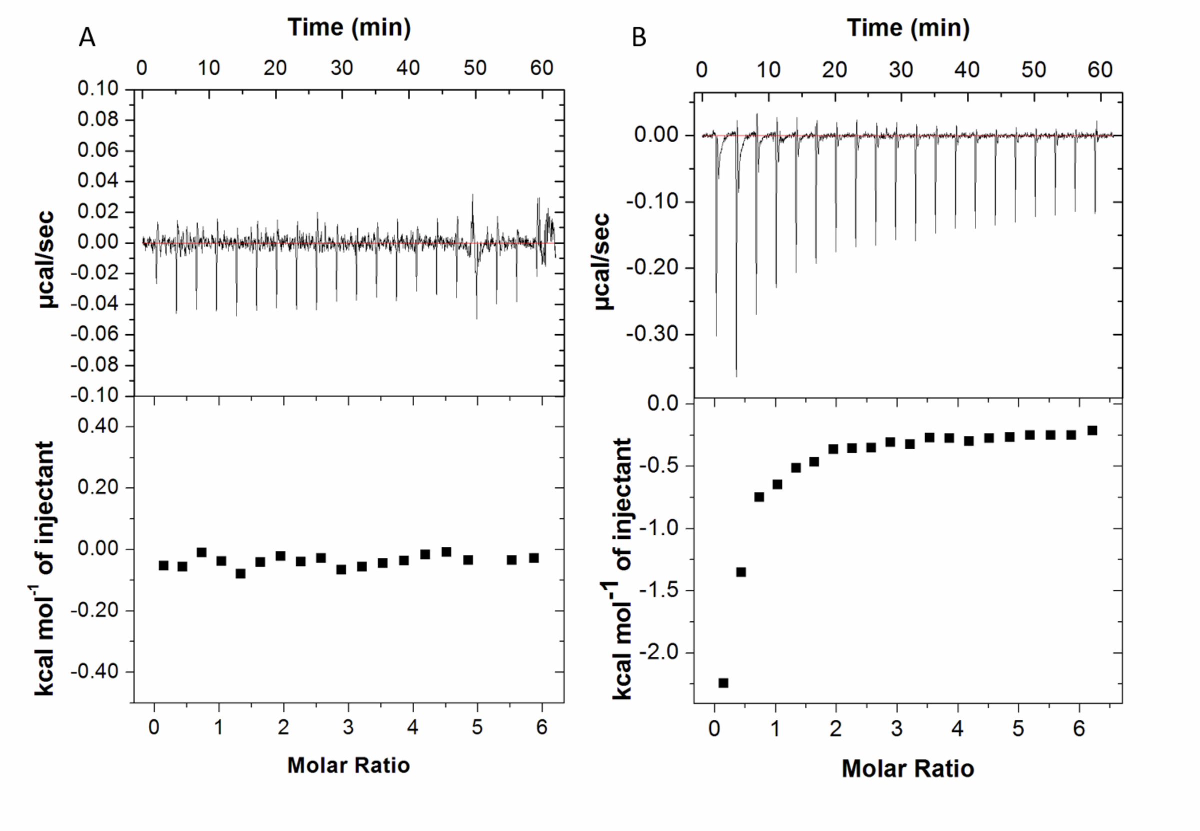

Figure S2 - ITC data of 50µM monomeric Ac αSyn with addition of A) arsenite ($NaAsO_2$) and B) cadmium ($CdCl_2$) at a final ratio of 1:6 monomers to metal ions. There's no quick binding to be detected for As(III). There is a significant heat exchange with the addition of Cd(II) that may suggest a weak binding to Ac αSyn assembled species.

References

1. Li, Y.; Zhao, C.; Luo, F.; Liu, Z.; Gui, X.; Luo, Z.; Zhang, X.; Li, D.; Liu, C.; Li, X. Amyloid fibril structure of α-synuclein determined by cryo-electron microscopy. *Cell Research* **2018**, *28*, 897-903, doi:10.1038/s41422-018-0075-x.
2. Guerrero-Ferreira, R.; Taylor, N.M.; Arteni, A.-A.; Kumari, P.; Mona, D.; Ringler, P.; Britschgi, M.; Lauer, M.E.; Makky, A.; Verasdonck, J.; et al. Two new polymorphic structures of human full-length alpha-synuclein fibrils solved by cryo-electron microscopy. *eLife* **2019**, *8*, doi:10.7554/elife.48907.
3. Li, B.; Ge, P.; Murray, K.A.; Sheth, P.; Zhang, M.; Nair, G.; Sawaya, M.R.; Shin, W.S.; Boyer, D.R.; Ye, S.; et al. Cryo-EM of full-length α-synuclein reveals fibril polymorphs with a common structural kernel. *Nature Communications* **2018**, *9*, doi:10.1038/s41467-018-05971-2.
4. Ni, X.; Mcglinchey, R.P.; Jiang, J.; Lee, J.C. Structural Insights into α-Synuclein Fibril Polymorphism: Effects of Parkinson's Disease-Related C-Terminal Truncations. *Journal of Molecular Biology* **2019**, *431*, 3913-3919, doi:10.1016/j.jmb.2019.07.001.
5. Zhao, K.; Li, Y.; Liu, Z.; Long, H.; Zhao, C.; Luo, F.; Sun, Y.; Tao, Y.; Su, X.-D.; Li, D.; et al. Parkinson's disease associated mutation E46K of α-synuclein triggers the formation of a distinct fibril structure. *Nature Communications* **2020**, *11*, doi:10.1038/s41467-020-16386-3.
6. Iyer, A.; Roeters, S.J.; Schilderink, N.; Hommersom, B.; Heeren, R.M.A.; Woutersen, S.; Claessens, M.M.A.E.; Subramaniam, V. The Impact of N-terminal Acetylation of α-Synuclein on Phospholipid Membrane Binding and Fibril Structure. *Journal of Biological Chemistry* **2016**, *291*, 21110-21122, doi:10.1074/jbc.m116.726612.
7. Boyer, D.R.; Li, B.; Sun, C.; Fan, W.; Sawaya, M.R.; Jiang, L.; Eisenberg, D.S. Structures of fibrils formed by α-synuclein hereditary disease mutant H50Q reveal new polymorphs. *Nature Structural & Molecular Biology* **2019**, *26*, 1044-1052, doi:10.1038/s41594-019-0322-y.
8. Sun, Y.; Hou, S.; Zhao, K.; Long, H.; Liu, Z.; Gao, J.; Zhang, Y.; Su, X.-D.; Li, D.; Liu, C. Cryo-EM structure of full-length α-synuclein amyloid fibril with Parkinson's disease familial A53T mutation. *Cell Research* **2020**, *30*, 360-362, doi:10.1038/s41422-020-0299-4.
